# Editorial: Objective dietary assessment in nutrition epidemiology studies, volume II

**DOI:** 10.3389/fnut.2026.1802043

**Published:** 2026-02-24

**Authors:** Amanda J. Lloyd, Thomas Wilson, Hariom Yadav

**Affiliations:** 1 Department of Life Science, Aberystwyth University, Aberystwyth, United Kingdom; 2 Department of Neurosurgery, Brain and Spine, University of South Florida, Tampa, FL, United States

**Keywords:** diet, diet recording, dietary assessment methodology, dietary assessment tools, dietary patterns, epidemiology studies, nutrition, population studies

## Introduction

Accurately capturing what people eat and drink remains one of the greatest challenges in public health nutrition and nutrition epidemiology. Despite decades of refinement, self-report tools, such as food frequency questionnaires, diet diaries, and recalls remain prone to recall bias, under- or over-reporting, and cultural variability. These limitations introduce systematic error into diet, disease relationships, complicating causal inference and undermining reproducibility.

The first volume of this Research Topic underscored the importance of developing and validating objective dietary assessment methods, including the use of biomarkers, digital technologies, and calibration models. Volume II builds on this momentum, bringing together 21 original contributions spanning multiple continents, dietary cultures, and methodologies. Collectively, these studies illustrate the richness of approaches being pursued: novel dietary indices, validation of culturally tailored tools, population-level surveillance, and integration of nutritional biomarkers with epidemiological outcomes.

## Dietary indices and chronic disease outcomes

A core theme in this volume is the use of dietary indices to capture overall diet quality or nutrient-specific exposures in relation to chronic disease outcomes.

Yu et al. assessed diet quality and quantity using the Chinese Diet Balance Index 2022, demonstrating that imbalances in diet quality increased the risk of sarcopenia. Their findings highlight the relevance of multidimensional diet quality indices in aging populations.

Wen et al. investigated the Composite Dietary Antioxidant Index (CDAI) in relation to growth indicators among children aged 3–12 years, showing that antioxidant-rich diets were associated with more favorable growth trajectories. Complementing this, Cheng et al. applied the CDAI to a U.S. pediatric population, revealing associations between antioxidant intake and susceptibility to Epstein–Barr virus infection, thus extending the relevance of CDAI beyond growth into infectious disease risk.

Zhou et al. explored the Oxidative Balance Score (OBS) and found significant associations with peripheral artery disease in U.S. adults, underscoring the role of diet-driven oxidative stress in vascular health. Yang S. et al. added to this theme by reporting that higher dietary phosphorus intake was protective against cardiovascular mortality among individuals with asthma, illustrating how nutrient-specific measures can be applied to at-risk clinical populations.

Altogether, these studies demonstrate the power of composite indices and targeted nutrient measures as standardized approaches to link diet with a spectrum of chronic health outcomes.

## Population-based studies on dietary patterns

A second cluster of papers examined dietary diversity, nutrient intake, and diet-related risks across diverse populations.

Qin et al. analyzed spatiotemporal trends in dietary diversity among Chinese residents, mapping both improvements and persistent regional disparities. Liang et al. focused on the association between retinol intake and hyperuricaemia in Southwest China, highlighting potential nutrient, disease links of public health concern. Xu et al. provided projections on the future burden of chronic kidney disease due to type 2 diabetes; attributable to dietary risks, drawing on Global Burden of Disease 2021 data, adding a valuable predictive dimension.

Alkazemi et al. investigated dietary magnesium and fiber intakes among women with metabolic syndrome in Kuwait, finding systematically low intake levels with clinical implications. Alemu et al. examined dietary diversity among pregnant women in Ethiopia, reporting suboptimal dietary adequacy with implications for maternal and child health. Mohammed et al. documented urban–rural disparities in minimum acceptable diet intake among Ethiopian children, while Endawkie et al. extended this by identifying household and community level factors underlying zero vegetable and fruit consumption in East Africa.

These analyses provide a picture of dietary diversity and risk factors across Asia, the Middle East, and Africa, illustrating the geographic breadth of challenges in dietary assessment and the need for culturally sensitive, region-specific dietary surveillance.

## Methodological validation and feasibility studies

Advances in dietary assessment methodology were another major theme of this Research Topic.

Saxby et al. piloted the feasibility of self-reported dietary recalls among newly diagnosed multiple sclerosis patients, demonstrating that dietary recalls remain practical, though they require adaptation to clinical contexts. Ni et al. developed and validated a food frequency questionnaire tailored for pregnant women from the Chinese Miao ethnic group, demonstrating both reproducibility and validity in a culturally specific population, a critical step toward equitable dietary research.

Ahern et al. analyzed secular trends in *ad libitum* energy intake in controlled research settings from 1999 to 2020, showing both consistency and shifts that underscore the importance of methodological calibration across decades of study designs.

Wardenaar et al. contributed two related studies in sports nutrition: first, the development of the Safe Supplement Screener (S3), designed to assess risk behavior in supplement use among athletes; and second, a cross-validation study of S3 in NCAA Division I athletes. Together, these papers highlight how structured, evidence-based screeners can enhance accuracy and safety in supplement reporting, where misclassification may have clinical and regulatory implications.

These methodological contributions collectively underscore the importance of ongoing validation, cultural tailoring, and integration of new tools into specific research and clinical populations.

## Nutritional status and special populations

Several contributions addressed dietary assessment in vulnerable or clinical populations where nutritional risks are acute.

Shifera and Yosef investigated undernutrition among tuberculosis patients in Ethiopia, identifying predictors that could inform targeted interventions. Takano et al. evaluated nutritional status and oral supplement use in elderly daycare users in Japan, leveraging a web-based version of the Mini Nutritional Assessment (MNA Plus) to demonstrate the potential of digital tools for real-world clinical screening.

Feng et al. examined dietary glycine intake in relation to hypertension, hyperlipidaemia, and obesity in rural northern China, providing novel evidence on amino acid intake and metabolic risk. Yang X. et al. conducted a global analysis of developmental and intellectual disabilities attributable to iodine deficiency from 1990 to 2019, with predictions to 2030, highlighting enduring global disparities in micronutrient deficiency.

These papers reinforce the crucial role of accurate, context-appropriate dietary assessment in vulnerable groups, where the stakes for mismeasurement are particularly high.

## Conclusion

The 21 contributions in Volume II collectively extend the field of dietary assessment in three critical ways. First, they demonstrate the expanding toolkit of indices, screeners, and validated questionnaires capable of capturing both nutrient-specific and overall dietary exposures. Second, they highlight the importance of context and population diversity, with studies spanning children, pregnant women, athletes, elderly populations, and patients with chronic or infectious diseases, across Asia, Africa and the Middle East. Finally, they highlight the integration of objective and semi-objective approaches, from biomarkers and antioxidant indices to digital tools and culturally specific FFQs as the path forward for more reliable nutritional epidemiology.

As dietary assessment continues to evolve, the integration of objective tools with culturally sensitive approaches will be essential for advancing global nutrition science.

Persistent challenges remain. These include the need to:

- Harmonize methods across cohorts to enable comparability.
- Reduce cost and participant burden to scale objective methods.
- Integrate self-report and objective measures through robust calibration.
- Ensure cultural adaptability and equity in dietary assessment tools.

The contributions in this volume demonstrate that the field is moving rapidly toward more precise, inclusive, and technologically enabled dietary assessment. By building on methodological innovation and global diversity, the work presented here brings us closer to resolving the long-standing limitations of dietary self-report and strengthening the evidence base for diet, disease relationships.

We thank all authors, reviewers, and the editorial team for their efforts, and we hope this Research Topic helps future innovation and collaboration, creating more accurate, equitable, and globally relevant dietary epidemiology.

